# PD-1, IL-10, IFN-γ and IL-12 form a network to regulate HIV-1-specific CD4 T Cell and antigen-presenting cell function

**DOI:** 10.1186/1742-4690-9-S2-O45

**Published:** 2012-09-13

**Authors:** F Porichis, L Barblu, DS Kwon, M Hart, J Zupkosky, GJ Freeman, DG Kavanagh, DE Kaufmann

**Affiliations:** 1 Ragon Institute of MGH, MIT and Harvard, Charlestown, MA, USA; 2 Dana-Farber Cancer Institute, Harvard Medical School, Boston, MA, USA

## Background

PD-1 and IL-10 blockade can restore antigen-specific T cell functions in chronic infections and cancer. However, not all subjects respond to inhibition of either pathway, the potential differences in functions restored by these interventions are unknown, and mechanistic interactions between these pathways are poorly understood.

## Methods

We investigated 45 subjects with HIV-1 infection with different disease status. We used CFSE assays to measure proliferation of HIV-1-specific CD4 T cells and Luminex arrays to analyze IFN-γ(Th1), IL-2(Th0), IL-13(Th2) and IL-12 secretion in supernatants of CD8-depleted PBMC stimulated for 48h with Gag peptide pools in the presence of isotype control antibody, anti-PD-L1 and/or anti-IL-10Ra, anti-IFN-γ or anti-IL-12. We used flow cytometry to evaluate the role of IFN-γ in regulating PD-L1, HLA-DR, HLA-ABC and CD86 expression by monocytes.

## Results

Whereas PD-L1 blockade had a balanced impact on proliferation and cytokine secretion by HIV-1-specific CD4 T cells, anti-IL-10Rα preferentially restored IFN-γ production. Combined blockade resulted in a dramatic 9.8-median-fold increase of IFN-γ, contrasting with the moderate effect of single blockade (2.5 median-fold). Antigenic stimulation of HIV-1-specific CD4 T cells upregulated PD-L1, HLA-DR and HLA-ABC on monocytes through an IFN-γ-dependent mechanism. Combined PD-L1/IL-10Rα induced a striking increase in IL-12 production by antigen-presenting cells(APCs) that was governed by IFN-γ derived from the Thelper cells. Neutralization of IL-12 reduced the dramatic effect of combined blockade on IFN-γ, demonstrating a positive feedback loop between IFN-γ produced by HIV-1-specific CD4 T cells and IL-12 produced by APCs.

## Conclusion

These data provide important evidence on the therapeutic potential of combined interventions on the PD-1 and IL-10 pathways to restore HIV-1-specific CD4 T cell and antigen-presenting cell function. We provide mechanistic insight on the mode of action of dual blockade by showing that IFN-γ produced by HIV-1-specific CD4 T-cells and IL-12 secreted by APCs regulate each other in a positive feedback loop.

